# The Herbal Constituents in An-Gong-Niu-Huang Wan (AGNH) Protect against Cinnabar- and Realgar-Induced Hepatorenal Toxicity and Accumulations of Mercury and Arsenic in Mice

**DOI:** 10.1155/2021/5566078

**Published:** 2021-04-01

**Authors:** Songsong Wang, Xiao Xiao, Ao Li, Peng Li

**Affiliations:** ^1^State Key Laboratory of Quality Research in Chinese Medicine, Institute of Chinese Medical Sciences, University of Macau, Macao 999078, China; ^2^College of Pharmacy and Bioengineering, Chongqing University of Technology, Chongqing 400054, China; ^3^Clinical Research Center, Affiliated Hospital of Guangdong Medical University, Zhanjiang 524001, Guangdong, China

## Abstract

An-Gong-Niu-Huang Wan (AGNH) has been a well-known cinnabar- and realgar-containing compound recipe for cerebral diseases. Unfortunately, its clinical practice is often restrained by the specific hepatorenal toxicity of cinnabar and realgar (C + R). In previous research studies, we have found that the antioxidative and anti-inflammatory effects of its herbal constituents could mitigate the risks from the toxicity. The underlying detoxification mechanisms are still unsolved. The present study investigated the protective effects of AGNH's herbal constituents on hepatorenal injury induced by C + R. For the mice treated with C + R, the increased expression levels of sensitive biomarkers of metal exposure and hepatorenal toxicity, including metallothionein (MT) in both hepatorenal tissues and kidney induced molecule-1 (KIM-1) in the kidney, were simultaneously reduced when C + R coadministered with other herbal medicines. In addition, the contents of trivalent As (As^III^), pentavalent As (As^v^), and mercury (Hg) in hepatorenal tissues of mice were also significantly reduced benefiting from the herbal constituents in AGNH. Further mechanism studies showed that the herbal constituents in AGNH could downregulate the expressions of uptake transporters (AQP9 and OAT1) and upregulate the expressions of efflux transporters (P-gp, MRP2, and MRP4) in mice intoxicated by C + R. Our results suggested that AGNH's herbal constituents protect the body against C + R-induced hepatorenal toxicity and accumulations of Hg and As, which could be associated with the reestablishment of heavy metal homeostasis and the detoxification system.

## 1. Introduction

An-Gong-Niu-Huang Wan (AGNH) has been a renowned compound recipe for treating cerebral diseases [1]. Mineral medicinal materials cinnabar and realgar (C + R) (with 96% of HgS and 90% of As_4_S_4_) are contained, accounting for 12.5% by weight in the formula [2]. In recent years, realgar-induced hepatic injury and cinnabar-induced renal toxicity have been reported [3–5], which are often associated with overdose or prolonged exposure. Furthermore, it is evident that AGNH contains multiple herbal ingredients, which are deemed to function in delivering drugs to target tissues as well as eliminating the harmful influences of the metallic ingredients including mercury (Hg) and arsenic (As) [6].

Various metabolizing enzymes and transporters mediate heavy metal compounds' metabolism and secretion. As a family of phase-2 detoxification enzymes in vivo, glutathione S-transferases (GST) could catalyze the conjugation of reduced glutathione (GSH) to mercuric and arsenical species [7, 8]. The generated Hg- and As- glutathione S-conjugates were transported across the canalicular membrane into the bile by the basolateral efflux transporters, such as P-glycoprotein (P-gp) and members of the multidrug resistance-associated protein family (MRP) [9]. These glutathione S-conjugates may also traverse the cell membrane at the apical end of renal proximal tubular epithelial cells (RPTECs) into urine assisted by the transporters akin to those expressed in hepatocytes [10]. On the other hand, drugs extracted from the blood into hepatocytes and renal tubular cells are mediated by basolateral uptake transporters, including organic anion and cation transporter (OAT/OCT) family members [11, 12]. The coordinated action of efflux and uptake transporters regulates the amount of mercuric and arsenical glutathione S-conjugates that transverse out of or return to hepatocytes and renal tubular cells. Our previous studies have shown that the combination of herbal medicine in AGNH can mitigate inflammation and injury in the liver and kidney tissues of mice induced by C + R [13, 14]. However, little is known about how AGNH's herbal ingredients act on phase-2 sulfotransferases and phase-3 uptake and efflux transporters induced by C + R.

In the present study, we wanted to compare the toxicity of AGNH with C + R in mice, focusing on the content of total As, water-soluble As including trivalent As (As^III^) and pentavalent As (As^v^) in sera, hepatorenal tissues, and the expressions of both metallothionein (MT) and kidney-induced molecule-1 (KIM-1), and sensitive biomarkers of metal exposure and hepatorenal toxicity. Furthermore, we further investigated whether the alleviating effects of other herbal medicines in AGNH to hepatorenal damage resulted from the overload of Hg and As are mediated, at least in part, by the drug-processing enzymes and transports in hepatorenal tissues of mice.

## 2. Materials and Methods

### 2.1. Chemicals and Animals

AGNH pill (weighted 3 g for each), cinnabar (containing 96% of HgS), as well as realgar (containing 90% of As_4_S_4_) were all provided by Guangzhou Bai-Yun-Shan Zhong-Yi Pharmaceutical Company Ltd. (Guangzhou, China). Methanol (Chromatographic grade) was purchased from Sigma Chemical Company (St. Louis, MO, USA), and ammonium carbonate (99.999%) was purchased from Aladdin Industrial Corporation (Shanghai, China). The As^III^, As^V^, and Hg standard solutions (1000 mg/L) were purchased from Sigma-Aldrich company, using as the standard curve solution. The production of ultrapure water (UPW) at 25°C depended on the Milli-Q® lab water purifying system (Merck, MA, USA). Specific antigens for the primary antibodies were as follows: GST*α*1, GSTmu, OCT1, OCT2, MRP1, aquaporin-9 (AQP-9), and *β*-actin (Bioworld Technology Co, Ltd., MN, USA); Na^+^-K^+^-ATPase, and MT-1 (Santa Cruz, CA, USA); KIM-1, GSTpi, MRP2, MRP4, P-gp, and OAT1 (Boster Biological Technology Co, Ltd., Hubei, China).

### 2.2. Animals and Experimental Design

Thirty-six Kunming (KM) mice (20 ± 2 g bodyweight, half of male and female) were provided by the Center of Experimental Animals of the Army military Medical University in Chongqing, China, and randomly assigned into 3 groups, with 12 in each group. All mice were housed in an environment with the controlled temperature of 22 ± 1°C and the humidity of 50 ± 2% under a 12 : 12 h light/dark (L/D) cycle. Before experiments, the mice were acclimatized to standard rodent diet and free access to water for 1 week. Mice were then treated with saline (vehicle), AGNH (2.5 g/kg), and equal amounts (0.14 g/kg + 0.14 g/kg) of C + R by intragastric administration once daily for 4 weeks, respectively. According to the Chinese Pharmacopoeia (2020 edition), the daily dosage of AGNH is 3 g/day for adults. In the present study, the dosage chosen for the mice (2.5 g/kg/day) is equal to five times the clinical equivalent dose. Since equal amounts (0.056 g) of cinnabar and realgar were contained in per gram AGNH, one group of mice was treated by oral administration of cinnabar (0.14 g/kg) and realgar (0.14 g/kg) per day for comparison. The details of the dosing regimen were described in our previous report [13, 14]. At 1 h after the last dosing, mice were anesthetized by pentobarbital sodium (50 mg/kg) intraperitoneally and sacrificed by cervical dislocation after collection of blood samples from the eyeballs. Hepatorenal tissues of mice were also collected and reserved for further analysis. All animal procedures were carefully approved by the Institutional Ethics Committee of the Chongqing University of Technology and performed in complete accordance with the National Institutes of Health Guidelines for the Care and Use of Laboratory Animals (8th Edition, 2011).

### 2.3. Analysis of the Speciation of As and the Content of Hg in the Sera and Hepatorenal Tissues of Mice

Serum samples (55 *μ*l) were added to 0.3 ml of mixture of methanol-water (*v*/*v*, 1 : 1), fully mixed using vortex for 30 sec. Subsequently, the mixture was extracted in the ultrasonic waterbath (10 min) and centrifugated (10 min, 15,000 g). Then, ion chromatography inductively coupled plasma mass spectrometry (IC-ICP-MS, Thermo Fisher Scientific, MA, USA) was applied for the analysis of As speciation in the supernatant of the mixture (180 *μ*l). The supernatant (150 *μ*l) left was diluted to 1.5 ml in purified water for the Hg content assay using the ICP-MS system (Thermo Fisher Scientific).

Hepatorenal tissues weighing 35 mg were mixed with 2 ml ultrapure water, followed by homogenization, bath in the ultrasonic waterbath (10 min) as well as centrifugation (15,000 g, 50 min, 4°C). The supernatant of the mixture was filtered through a 0.22 *μ*m polytetrafluoroethylene (PTFE) membrane prior to IC-ICP-MS and ICP-MS analyses.

The ICP-MS (ICAP-Q) was programmed to detect the speciation of As and the content of Hg in our study, mainly including the parameters of forward radiofrequency (RF) power (1550 W), auxiliary gas flow rate of argon (12.9 l/min), gas flow rate of nebulizer argon (0.70 l/min), and dwell time (200 ms). Chromatographic separation of As speciation was carried out using the Thermo Scientific Dionex ICS-5000 IC system coupled with Thermo Scientific Dionex IonPac AS7 Specialty Anion-Exchange (AE) Column (4 × 250 mm for pore size, 10 *μ*m for bead diameter). The mobile phase was composed of ammonium carbonate (mobile phase A, 5 mM) and ammonium carbonate (mobile phase B, 200 mM). Besides, the gradient elution system was programmed as 0% B for 0–2.0 min, 0–100% B for 2.0-2.1 min, 100% B for 2.1–5.5 min, 100–0% B for 5.5-5.6 min, and 0% B for 5.6–10 min, setting the flow rate of the mobile phase to 1.0 ml/min as well as the injected sample volume to 25 *μ*l. In addition, linearity, accuracy, precision, LOD (limit of detection), and LOQ (limit of quantitation) of IC-ICP-MS were fully verified by characteristic indicators.

### 2.4. Immunohistochemical Examination

Paraffin-embedded sections of hepatorenal tissues were dewaxed with xylene and hydrated with gradient ethanol. For antigen retrieval, the slices were then put into a boiled citrate buffer for 20 min (pH 6.0, 10 mM). Afterwards, H_2_O_2_ of 3% (*v*/*v*) was adopted for the inactivation of endogenous enzymes such as peroxidase. Subsequently, sections were probed with the primary antibody (dilution, 1 : 150) against MT-1 or KIM-1 overnight at 4°C. Secondary antibody incubation was performed with a Polink-2 polymer conjugated with horseradish peroxidase (HRP, Beijing Zhongshan Golden Bridge Biotechnology Co., Ltd., Beijing, China). Positive reactions in 3, 3′-diaminobenzidine- (DAB-) stained areas were then visualized. Hematoxylin was further adopted for counterstaining the nuclei. Images for immunohistochemistry staining (*n* = 5 per mice) were acquired on a bright-field Olympus microscope (BX51) at a magnification of ×200. The expression levels of MT-1 and KIM-1 were quantified using the integrated optical density (IOD) for immunoreactive regions. Five random fields were collected from each section, and their IOD values were determined by the image analysis software of Image-Pro ^®^ Plus (Version 6.0, Media Cybernetics Inc., USA).

### 2.5. Western Blotting (WB) Analysis

The extraction of the total protein from hepatorenal tissues of mice was performed using RIPA buffer (Beyotime Biotechnology, Jiangsu, China) plus protease inhibitors (Solarbio, Beijing, China). Then, the bicinchoninic acid (BCA) protein assay was available in kit (Beyotime) to further quantify the concentrations of protein. Proteins (30 *μ*g) were resolved by sodium dodecyl sulfate polyacrylamide gel (SDS-PAGE) electrophoresis and transblotted to PVDF membranes, which were subsequently blocked with skimmed milk (5% *w*/*v*) in Tris‐buffered saline with Tween 20 (TBST) for 1 h. The desired primary antibodies against KIM-1, MT-1, GST*α*1, GSTmu, GSTpi, OCT1, OCT2, MRP1, MRP2, MRP4, P-gp, OAT1, AQP-9, and *β*-actin were probed onto the membranes overnight at the temperature of 4°C. After washing in TBST, membranes were further incubated for 1 h with specific secondary antibodies at the dilution of 1 : 10000 (Bioworld). Besides, *β*-actin or Na^+^-K^+^-ATPase was referred as an loading control. An enhanced chemiluminescence kit (Millipore, MA, USA) was adopted for the visualization of immunoprobed proteins. The capture of immunosignals depended on Amersham™ Imager 600 (Amersham Biosciences-GE Healthcare, UK). In addition, the quantification of band intensities was performed using the software of Quantity One for WB analysis (Version 4.62, Bio-Rad Laboratories, Inc., USA). Defining the level in the saline control group as 1-fold, the variations in the band intensity were presented as changes of fold.

### 2.6. Statistical Analysis

The statistical analysis was conducted with IBM^®^ SPSS^®^ statistical software package for Windows (Version 18.0, Chicago, IL, USA). Values for continuous variables were described as mean ± standard deviation (SD). One-way analysis of variance (ANOVA) was used to compare the significance of variation among the 3 groups, applying *P* < 0.05 as the level of statistical significance in all cases.

## 3. Results

### 3.1. Toxicity-Related Protein Expressions in Hepatorenal Tissues of Mice

**Figure 1:**
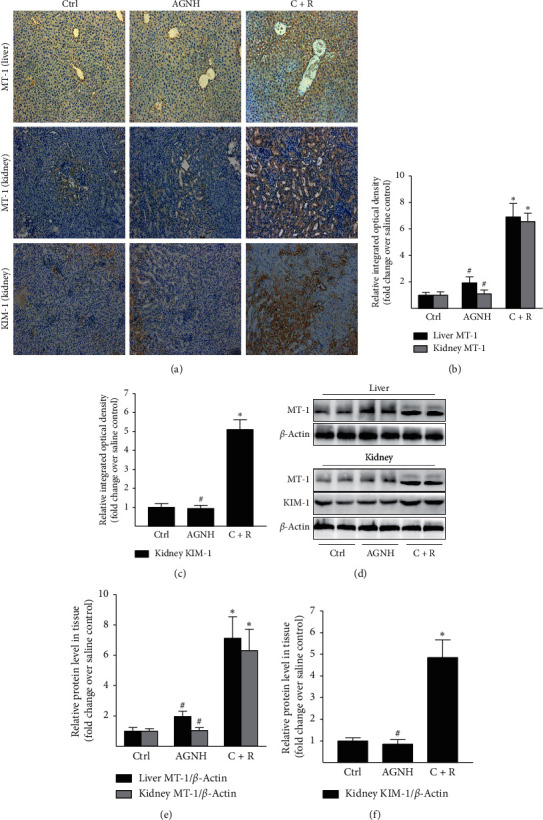
AGNH's herbal constituents alleviate hepatorenal impairment induced by cinnabar and realgar (C + R) in mice. Mice received saline (vehicle), AGNH (2.5 g/kg), as well as C + R (0.14 g/kg + 0.14 g/kg) orally once daily for 4 weeks, respectively. (a) Representative immunostaining images of MT-1 and KIM-1 in the hepatic and renal sections of mice. Scale bar: 100 *μ*m. (b), (c) Semiquantitative immunohistochemical analysis of MT-1 and KIM-1 in the respective group. (d) WB analysis of MT-1 and KIM-1 levels in the hepatorenal tissues. The level of housekeeping protein *β*-actin was referred as a control for equal protein loading. (e), (f) Band intensities were converted to arbitrary densitometric units (ADUs), normalized by the value of *β*-actin, and finally expressed according to the levels in saline-treated mice (defined as 1-fold). Each bar represents mean ± standard deviation (SD) from six mice.  ^*∗*^*P* < 0.05 compared to the saline control group;  ^#^*P* < 0.05 compared to the C + R coadministration group.

Hepatorenal tissues are the major target tissues of As and Hg toxicity. MT plays a key part in the detoxification of As and Hg, and therefore, this protein is often considered to be one of the sensitive biomarkers to evaluate the injury to the hepatorenal tissues [15]. Immunohistochemical analysis showed both hepatocytes and RPTECs stained strongly positive for MT-1 when mice were treated with cinnabar combined with realgar for 28 days (Figures 1(a) and 1(b)), whereas positive staining for MT-1 was weak in the sections of hepatorenal tissues in mice treated with saline or AGNH. Besides, results obtained from WB analysis also revealed that MT-1 expression levels in hepatorenal tissues of mice were unchanged between groups of AGNH and saline (Figures 1(d) and 1(e)), but combined administration of C + R exerted a significant increasing effect on MT-1 protein levels in hepatorenal tissues of mice.

KIM is another biomarker whose expression is highly increased in the renal proximal tubule in mice after kidney injury [16]. By immunohistochemical staining, almost all RPTECs from mice coexposed to C + R were strongly stained positive for KIM-1 (Figures 1(a) and 1(c)); whereas, few KIM-1-positive RPTECs were found in the mice groups of saline and AGNH. By WB analysis, we also found that compared to that of the saline group, exposed to C + R markedly increased the KIM-1 expression level in the kidney of mice (Figures 1(d) and 1(f)); whereas, no significant difference of KIM-1 expression was detected between the mice treated with AGNH and those treated with saline.

### 3.2. As and Hg Quantification in the Sera, Liver, and Kidneys of Mice

As^III^ and As^V^ are well separated by optimization of mobile phase and gradient, and representative ion chromatograms of As speciation in the sera, liver, and kidneys of mice are shown in Supplemental Figure 1. Linearity, *R*^2^ and linearity range, LOD, LOQ, precision, and the accuracy of the IC-ICP-MS method for the analysis of As^III^ and As^V^ were tested. The precision of IC-ICP-MS was tested by relative standard deviation (RSD) in percentage. Detailed information is shown in Supplemental Table 1.

**Figure 2:**
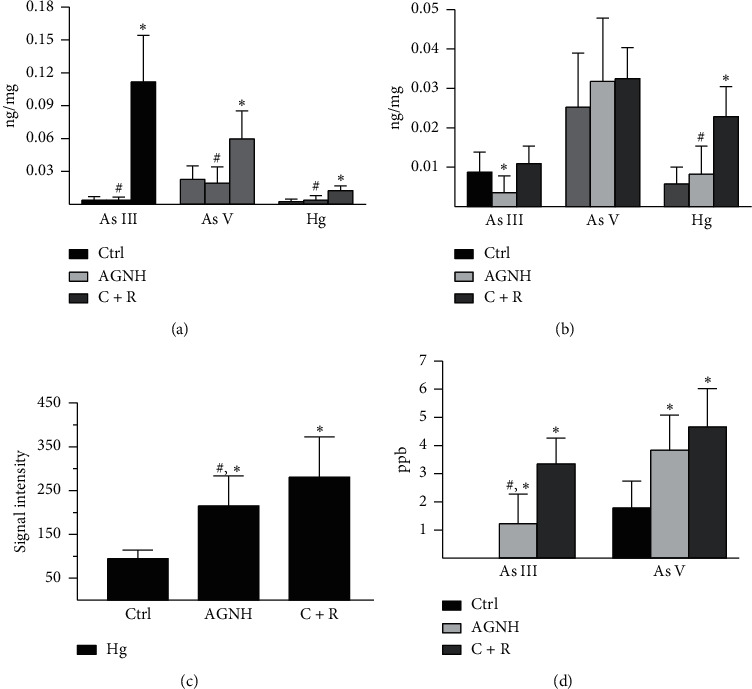
AGNH's herbal constituents prevented Hg and As accumulation induced by C + R in the hepatorenal tissues and sera of mice. Mice received saline (vehicle), AGNH (2.5 g/kg), as well as C + R (0.14 g/kg + 0.14 g/kg) orally once daily for 4 weeks, respectively. (a) Hepatic and (b) renal tissues from mice were obtained on day 28. As (As^III^ and As^V^) and Hg contents in the hepatorenal tissues from mice were analyzed by IC-ICP-MS. (c) Blood specimens from mice were also obtained on day 28. The signal intensity of Hg in the sera from mice was measured by ICP-MS. (d) As (As^III^ and As^V^) contents in the sera from mice were analyzed by IC-ICP-MS. Values for continuous variables are described as mean ± standard deviation (SD), *n* = 12.  ^*∗*^*P* < 0.05 compared to the saline control group;  ^#^*P* < 0.05 compared to the C + R coadministration group.

As shown in Figure 2(a), hepatic Hg content reached 0.12 ng per mg liver in the C + R group when compared to 0.004 ng per mg liver in the AGNH group, which was slightly higher than the saline control group (0.002 ng per mg liver). Similar results were obtained with the As^III^ and As^V^ content assay in the liver of mice, where the As^III^ and As^V^ contents in the C + R coadministrated mice were found to be significantly increased when compared with the saline-treated group, whereas there was no difference with statistical significance in contents of both As^III^ and As^V^ between the mice treated with AGNH and those treated with saline.

Renal Hg content in the mice after C + R coadministration was 3-4 folds higher than those in the mice treated with vehicle and AGNH (Figure 2(b)). Interestingly, the content of As^III^ in the kidneys of mice was even less in the group treated with AGNH than in the group treated with saline. Moreover, no obvious difference was detected concerning As^V^ content in the kidneys among the groups of mice.

Since Hg of the sera could be detected only in trace amount, we selected signal intensity from ICP-MS to evaluate the differences in Hg content between different groups. Results showed that the average signal intensity of sera from C + R cotreated mice was 2.9 times higher than that from the saline control group (Figure 2(c)). Coadministration of other herbal medicines with C + R in AGNH led to signal intensity that was only 76.70% of the intensity from C + R cotreated mice, despite having stronger signal intensity than that from the saline control group. Besides, similar results were discovered in the As^III^ and As^V^ content assay (Figure 2(d)).

### 3.3. Phase-2 Conjugation Enzyme and Transporter Expressions in the Hepatorenal Tissues of Mice

**Figure 3:**
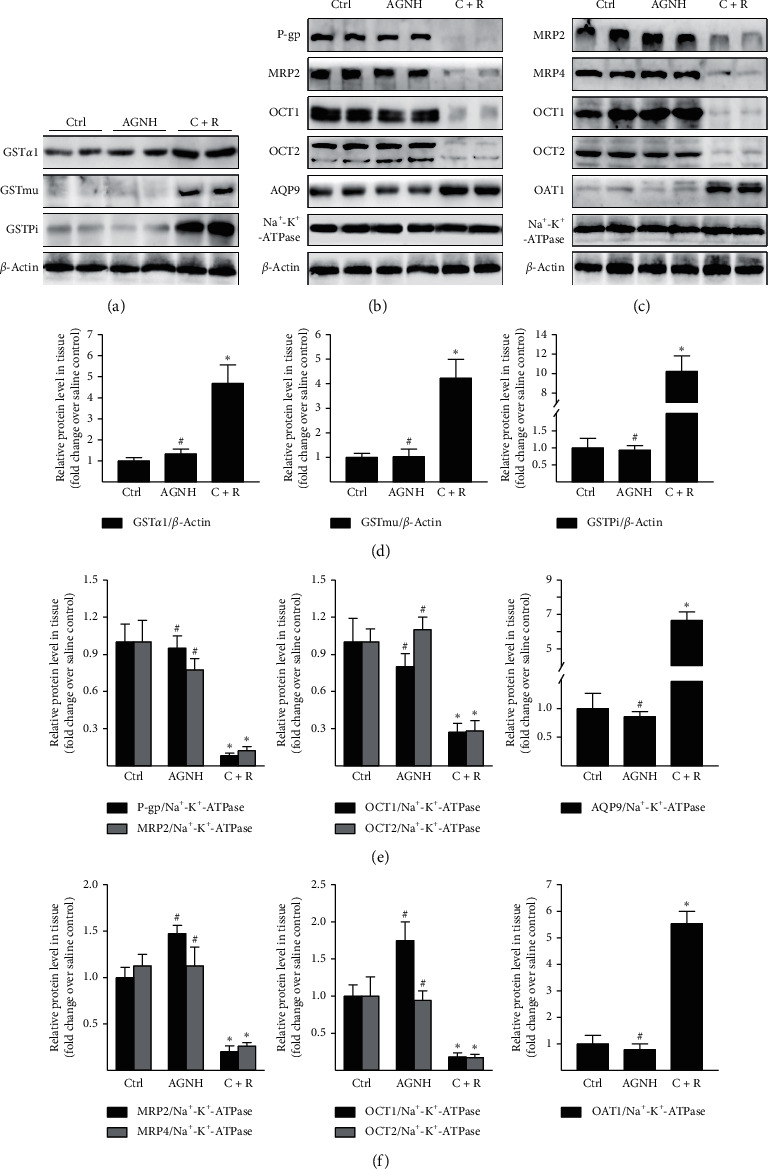
AGNH's herbal constituents inhibited expressions of GST and uptake transporters induced by C + R but enhanced the expressions of efflux transporters in the hepatorenal tissues of mice. Mice received saline (vehicle), AGNH (2.5 g/kg), as well as C + R (0.14 g/kg + 0.14 g/kg) orally once daily for 4 weeks, respectively. Hepatorenal tissues from mice were obtained on day 28. WB analysis of GST-*α*1, GSTmu, GSTpi, P-gp, MRP2, MRP4, OCT1/2, AQP9, and OAT1 in (a, b) hepatic and (c) renal tissues. The protein level of *β*-actin or Na^+^-K^+^-ATPase was referred as a loading control. (d–f) Band intensities were measured in arbitrary densitometric units (ADUs), normalized by the value of *β*-actin or Na^+^-K^+^-ATPase and finally expressed according to the levels in saline-treated mice (defined as 1-fold). Values for continuous variables are described as mean ± standard deviation (SD), *n* = 6.  ^*∗*^*P* < 0.05 compared to the saline control group;  ^#^*P* < 0.05 compared to the C + R coadministration group.

The potential detoxification mechanisms involved in metal-herb interactions in AGNH are further explored. GST catalyzes the conjugation of GSH with Hg and As and convert them to water-soluble glutathione S-conjugates, which are easily excreted to the bile with the help of the basolateral efflux transporters, including MRP2 and P-gp. The Hg- and As-glutathione S-conjugates may also traverse the luminal plasma membrane of RPTECs into urine by MRP2 and 4 [17]. WB analysis revealed that compared with the saline group, coadministration of C + R upregulated the expressions of GST family members (GST*α*1, GSTmu, and GSTpi) in the livers of mice (Figures 3(a) and 3(d)). In contrast, expressions of efflux family proteins including P-gp and MRP2 in the livers (Figures 3(b) and 3(e)) and MRP2 and 4 in the kidneys were downregulated after treating mice with C + R (Figures 3(c) and 3(f)). However, no significant differences in the expressions of GST and efflux family members were detected between the mice groups treated with AGNH and saline.

AQP9, an aquaglyceroporin protein, facilitates hepatic uptake of As from the portal blood and accelerates hepatocellular injury in the liver [18]. Furthermore, basolateral uptake transporters at RPTECs, including the members of OAT/OCT, extract the Hg-glutathione S-conjugates from the bloodstream, causing renal tubular injury [19]. WB analysis revealed that compared with the saline control group, C + R coadministration induced a marked increase in the protein expressions of AQP9 in the livers and OAT1 in the kidneys of mice (Figures 3(b), 3(c), 3(e), and 3(f)). Unexpectedly, C + R coadministration caused an obvious decrease in the protein expressions of OCT1 and 2 in the hepatorenal tissues of mice (Figures 3(b), 3(c), 3(e), and 3(f)). However, the protein expressions of AQP9, OAT1, OCT1, and OCT2 did not differ significantly between the mice groups treated with AGNH and saline.

## 4. Discussion

Our previous study has shown that the hepatorenal toxicity induced by C + R could be mitigated when used in combination with other herbs in AGNH [13]. Consecutive 28-day intragastric administration of C + R merely increased the aspartate aminotransferase (AST) activity as well as the alanine aminotransferase (ALT) activity in liver homogenates. However, it did not significantly influence blood biochemical parameters, suggesting routine serum biochemical markers may not be sensitive indices of hepatorenal impairment induced by C + R [14]. MTs are a family of cysteine-rich intracellular proteins with a well-known metal-binding ability that participate in heavy metal detoxification. MTs have been widely used as specific biomarkers reflecting heavy metal-induced tissue injury [20]. In addition, KIM-1 is a reliable histological biomarker to detect the progression of renal tubular injury [16]. Currently, compared with the saline group, MT-1 expressions in hepatorenal tissues and KIM-1 expression in the kidney were significantly higher in the C + R group, while levels of MT-1 and KIM-1 protein were similar after treatment with AGNH to the levels after coexposed to vehicle alone. The aforementioned studies further confirmed that the combined use of other herbal medicine in AGNH could attenuate the hepatorenal damage induced by C + R.

It is generally assumed that the hepatorenal toxicity induced by C + R relies on the amount and the diverse chemical forms of heavy metals in the target tissues; we next examined the As and Hg accumulation in the blood and hepatorenal tissues of mice. Treatment of C + R increased As (As^III^ and As^V^) and Hg levels in the liver, suggesting cinnabar- and realgar-induced hepatotoxicity may be initiated by As (As^III^ and As^V^) and Hg deposition. The changes of the As (As^III^ and As^V^) level in the kidney were different from those in the liver. Although C + R failed to increase the level of As (As^III^ and As^V^), this does not exclude the possibility that the oxidase and methylase in the kidney catalyzed the ingested As^III^ and As^V^ to form monomethyl arsenic acid (MMA^V^ and MMA^III^) and dimethyl arsenic acid (DMA^V^), which could be important sources of chronic nephrotoxicity in mice intoxicated by C + R [21]. In contrast, compared with the saline control group, AGNH, an Hg and As-containing traditional Chinese compound prescription, did not induce any significant Hg and As accumulation in hepatorenal tissues, suggesting that other herbal constituents in AGNH might promote Hg and As elimination from the hepatorenal tissues of mice. Further studies in future are necessary for exploring the potential mechanisms.

GST is an important phase-2 drug-metabolizing enzyme, which conjugates Hg and As with GSH to accelerate their biliary excretion [7]. The current study found that coadministration of C + R potently upregulated the hepatic expression of GST*α*1, GSTmu, and GSTpi, while treatment of AGNH had little effect on the expression of GST family members. This increase in the GST protein level facilitates biliary excretion of Hg and As-GSH conjugates, which might be an adaptive mechanism aimed at protecting the body against Hg and As overload in hepatocytes.

Xenobiotic transporters in the hepatorenal tissues have been found to be essential for the bile/urinary secretion and reabsorption of heavy metals [22]. As one of the most predominant mechanisms of Hg and As accumulation in hepatorenal tissues, C + R coadministration reduced Hg and As excretion via urine and bile, as reflected by a simultaneous decrease in the expressions of efflux transporters (P-gp, MRP2, and MRP4) and increase in the expressions of uptake transporters (AQP9 and OAT1), relative to those in saline-treated mice. However, no significant difference between the mice groups treated with AGNH and saline was discovered. There have been numerous reports that multiple herbal constituents in AGNH, such as curcumin, baicalin, berberine, and quercetin, possess a wide range of pharmacological activities including anti-inflammatory, antioxidative, and detoxifying activities [23–26]. In addition, they could interfere with the hepatobiliary and urinary elimination of drugs through affecting the major uptake and efflux transporters [23, 27–30]. Thus, it is reasonable to speculate that herbal constituents in AGNH could alleviate hepatorenal toxicity induced by C + R, probably by the decreased reabsorption and enhanced excretion of Hg and As in hepatorenal tissues.

## 5. Conclusion

In short, this study demonstrates that AGNH's herbal constituents protect against the accumulations of Hg and As and hepatorenal toxicity induced by C + R by decreasing expressions of uptake transporters and increasing expressions of efflux transporters in the hepatorenal tissues. These findings provide the first detailed detoxification metabolism regarding the herbal constituents in AGNH that protect from hepatic and renal injuries initiated by Hg and As deposition. Notably, blood Hg and As levels were significantly increased in the AGNH group than in the saline group. Thus, long-term consumption of AGNH at the higher dose should be considered with caution.

## Data Availability

The data used to support the findings of this study are included within the article.

## Supplementary Materials

Supplementary Materials Supplementary material includes methodology validation of AsIII, AsV, and Hg detection in Supplemental Table 1 and representative ion chromatograms of arsenic speciation in the sera, liver, and kidneys from mice administered by oral gavage with An-Gong-Niu-Huang Wan in Supplemental Figure 1.
