# Supplementary material for: The Herbal Constituents in An-Gong-Niu-Huang Wan (AGNH) Protect against Cinnabar- and Realgar-Induced Hepatorenal Toxicity and Accumulations of Mercury and Arsenic in Mice
Source: Evid Based Complement Alternat Med. 2021 Apr 1;2021:5566078. doi: 10.1155/2021/5566078 (PMC8035015; doi:10.1155/2021/5566078)
Supplement: Supplementary Materials — Supplementary material includes methodology validation of AsIII, AsV, and Hg detection in Supplemental Table 1 and representative ion chromatograms of arsenic speciation in the sera, liver, and kidneys from mice administered by oral gavage with An-Gong-Niu-Huang Wan in Supplemental Figure 1. [file 5566078.f1.docx]

**Supplemental Table 1** [**Methodology**](file:///C:\Users\Wss\AppData\Local\youdao\dict\Application\7.5.0.0\resultui\dict\?keyword=methodology)[**validation**](file:///C:\Users\Wss\AppData\Local\youdao\dict\Application\7.5.0.0\resultui\dict\?keyword=validation) **of As^III^, As^V^ and Hg detection**

| Species of heavy metals | Linearity | R² | Linearity range (μg/L) | LOD  (μg/L) | LOQ  (μg/L) | Recovery (%) | RSD (%) |
| --- | --- | --- | --- | --- | --- | --- | --- |
| As^III^ | y=17618x+ 457.6 | 0.999 | 0.05-2.0 | 0.02 | 0.05 | 78.7±6.9 ^a^  77.8±6.6 ^b^  83.3±3.8 ^c^ | 10.13^a^  8.91^b^  4.64 ^c^ |
| As^V^ | y=26419x+ 1616.0 | 0.996 | 0.05-2.0 | 0.02 | 0.05 | 93.6±5.0 ^a^  94.3±6.2 ^b^  92.0±2.7 ^c^ | 5.90^a^  6.30^b^  3.44^c^ |
| Hg | y=6415.x + 191.4 | 0.999 | 0.60-5.0 | 0.10 | 0.60 | 78.7±6.5^a^  79.1±4.8 ^b^  83.9±6.4 ^c^ | 8.21 ^a^  7.83^b^  8.80 ^c^ |

^a^Recovery and RSD in the liver.

^b^Recovery and RSD in the kidney.

^c^Recovery and RSD in the sera.

**
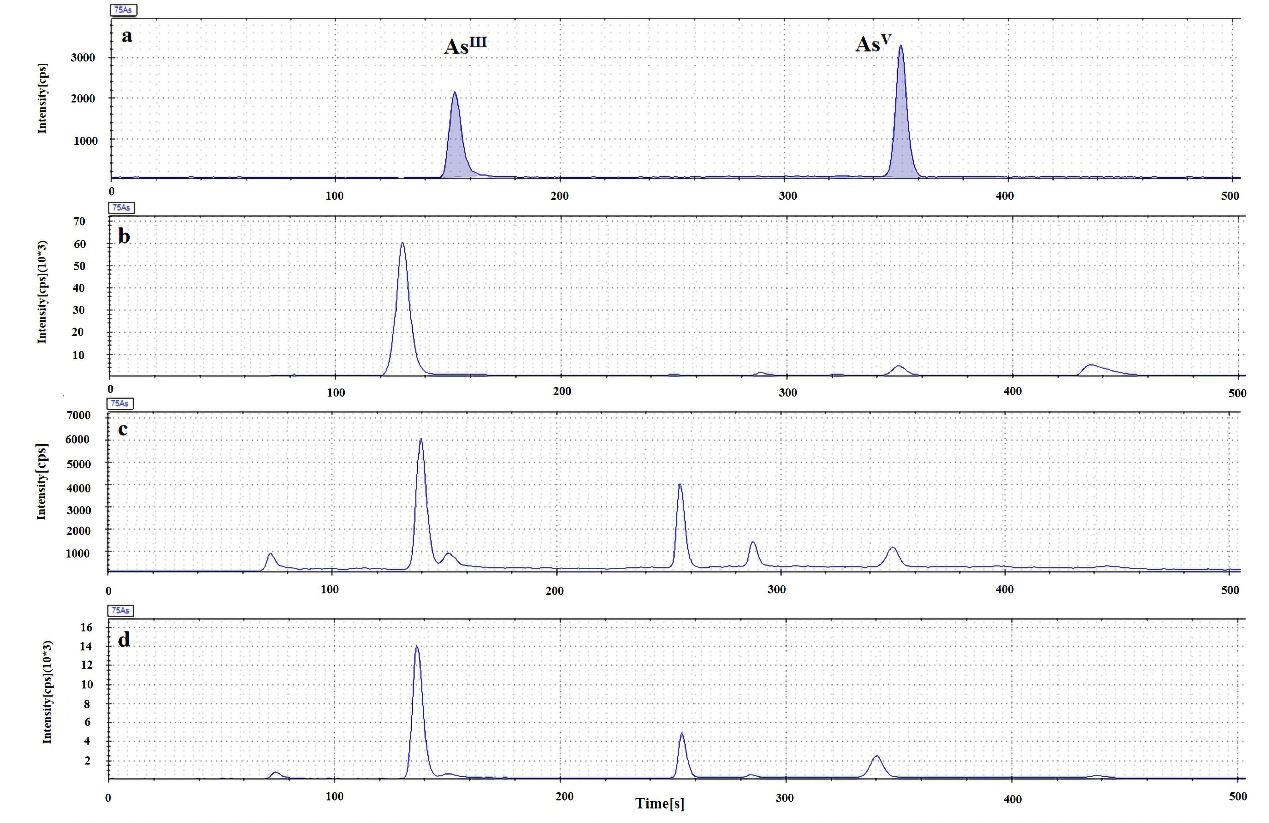
**

**Supplemental Figure 1** Representative ion chromatograms of arsenic speciation in the sera, liver, and kidneys from mice administered by oral gavage with An-Gong-Niu-Huang Wan (AGNH; 2.5 g/kg/d) once daily for 28 days. a. The ion chromatogram of mixture of standard reference of As^III^ and As^V^. b. The ion chromatogram of As^III^ and As^V^ in the sera. c. The ion chromatogram of As^III^ and As^V^ in the liver. d. The ion chromatogram of As^III^ and As^V^ in the kidney.
